# Clinical Diagnosis and Treatment of Chronic Pain

**DOI:** 10.3390/diagnostics13243689

**Published:** 2023-12-18

**Authors:** Sadiq Rahman, Ali Kidwai, Emiliya Rakhamimova, Murad Elias, William Caldwell, Sergio D. Bergese

**Affiliations:** Department of Anesthesiology, Stony Brook University Hospital, Stony Brook, NY 11794, USA; sadiq.rahman@stonybrookmedicine.edu (S.R.); ali.kidwai@stonybrookmedicine.edu (A.K.); emiliya.rakhamimova@stonybrookmedicine.edu (E.R.); murad.elias@stonybrookmedicine.edu (M.E.); william.caldwell@stonybrookmedicine.edu (W.C.)

**Keywords:** chronic pain, pain diagnostics, pain classification, pain approach, biopsychosocial

## Abstract

More than 600 million people globally are estimated to be living with chronic pain. It is one of the most common complaints seen in an outpatient setting, with over half of patients complaining of pain during a visit. Failure to properly diagnose and manage chronic pain is associated with substantial morbidity and mortality, especially when opioids are involved. Furthermore, it is a tremendous financial strain on the healthcare system, as over USD 100 billion is spent yearly in the United States on healthcare costs related to pain management and opioids. This exceeds the costs of diabetes, heart disease, and cancer-related care combined. Being able to properly diagnose, manage, and treat chronic pain conditions can substantially lower morbidity, mortality, and healthcare costs in the United States. This review will outline the current definitions, biopsychosocial model, subclassifications, somatosensory assessments, imaging, clinical prediction models, and treatment modalities associated with chronic pain.

## 1. Introduction

Chronic pain has become a ubiquitous condition encountered by many patients seeking medical care. Pain is a subjective experience and one of the leading causes of patient suffering and disability [1]. The Global Burden of Disease (GBD) Study highlighted the high prominence of pain as a global cause of disability in both developed and developing nations. The study also highlighted how this assessment of burden is also an underestimate. Chronic lower back pain, for example, was noted to be the single greatest cause of years lived with disability (YLD) around the world [2]. Lower back pain remains the leading cause of disability globally, impacting 619 million individuals globally in 2020, and it is anticipated to increase to 843 million in the next three decades [3].

Many forms of chronic pain, like lower back pain, come without a diagnosable underlying pathology and are often categorized as “non-specific” [4]. This has led to calls for research efforts to better understand the underlying pathophysiology of various chronic pain conditions. Other conditions of perceived pain, like central pain amplification, cannot currently be explained through somatic or neuropathic processes and are instead due to alterations in pain modulatory pathways [5].

“Non-specific” forms of chronic pain can lead to the pooling of heterogenous disease processes and consequently less specific pain management modalities. For optimized management of chronic pain, proper diagnosis and classification is highly recommended. This review will also discuss the recent somatosensory assessments, imaging, and clinical prediction models used for chronic pain conditions. The biopsychosocial treatment of chronic pain is also complex and multifaceted, including pharmacological interventions, physical rehabilitation, and interventional procedures [6,7,8].

## 2. Defining Chronic Pain

The most common symptom reported to health care providers is pain. Chronic pain is often used as an umbrella term for a wide range of painful conditions from nonspecific lower back pain to fibromyalgia to complex regional pain syndrome (CRPS). While acute pain may serve an adaptive role, chronic pain has been widely considered to be clinically maladaptive, that “neither protects nor supports healing and repair” [9]. Chronic pain was previously defined as pain persisting past a normal healing time and lacking the advantage of acute pain’s warning function [10].

It is often defined as pain that persists longer than “normal healing” and widely agreed to be at least three months in duration. Chronic pain also has been used as a label for a patient’s condition when underlying causes of pain are unclear or have been unidentified. This reinforces the need for more precise and updated methods of diagnosis and treatment for the many patients who encounter chronic pain.

The International Association for the Study of Pain (IASP) characterizes pain as an unpleasant sensory and emotional experience associated with, or resembling, actual or potential tissue damage [11]. The IASP definition of chronic pain has become widely adopted by health care professionals and academic researchers and even adopted by professional organizations such as the World Health Organization (WHO). 

The need for adequate revision for chronic pain diagnoses in the International Classification of Diseases (ICD) system has also been expressed as it is crucial for not only improvements in treatment but also for launching relevant research programs. Current iterations and classifications of chronic pain conditions are sometimes poorly defined and arbitrarily distributed in the ICD system [12]. As recently as the release of the ICD-10, chronic postsurgical and posttraumatic pain was not being represented; it was only defined as of 2022 within the ICD-11 [13].

Thoughts, emotions, and stress also affect the perception of pain, so a biopsychosocial assessment of pain helps provide a more complete definition and overview of conditions associated with chronic pain [14]. Neuroscience research indicates pain pathways in the central nervous system (CNS) often work in conjunction with emotions. Pain pathways can also be stimulated by peripheral tissues and traumatic experiences [15].

The biopsychosocial model views illness as a complex interaction between psychological, social, and biological factors [16]. This has also led to the development of an interdisciplinary pain management approach. Conceptualizing, assessing, and treating chronic pain would be incomplete without a sophisticated understanding of the emotional states and processes linked with the condition [17].

## 3. Burdens of Chronic Pain

Chronic pain does not come without significant personal and economic burden across the globe; affecting more than thirty percent of the world population [18]. Prevalence in the United States has been noted to vary between 11% and 40% with the US Centers for Disease Control and Prevention (CDC) estimating point prevalence at 20.4% (approximately 50 million), with higher prevalence rates associated with women and adults living in poverty and from lower socioeconomic backgrounds and rural areas [19]. Furthermore, the CDC estimates that 8% of U.S. adults (20 million) suffer high-impact chronic pain (i.e., interfering with work or life most days or every day).

In one cohort study [20], 61.4% of adults with chronic pain in 2019 had their condition continue in 2020 and estimated a higher incidence of chronic pain (52.4 cases per 1000 PY) compared to diabetes (7.1 cases per 1000 PY), depression (15.9 cases per 1000 PY), and hypertension (45.3 cases per 1000 PY). A strong example of the prevalence of chronic pain can be seen with lower back pain as it is a very common symptom experienced by patients of all ages [21,22,23].

Furthermore, chronic pain affects more than 50% of the older population and up to 80% of nursing homes residents [24]. Hyperalgesia and delayed recovery from pain secondary to nerve injury was also observed in this population [25]. Another novel finding in chronic pain research found high comorbidities with insomnia. Approximately 50% of chronic patients experience clinically significant sleep disturbances [26]. A lack of restorative sleep can further exacerbate and continue a cycle of chronic pain related symptoms.

Indicators of socioeconomic status including poverty, education, and health insurance coverage are associated with the presence of specific health conditions like chronic pain. It is estimated that chronic pain has an economic impact estimated to be USD 560 billion in direct medical costs, lost productivity, and disability [27]. Chronic pain also has a functional impact as one study found patients with chronic pain had increased difficulty with activities of daily living (21.5% vs. 4.9%), social engagement (25.4% vs. 5.7%), and work limitations (48.8% vs. 15%), leading to an estimated USD 79.9 billion in lost wages [28]. Effective diagnosis and treatment of chronic pain can help alleviate some of the financial impact chronic pain has on many households and communities.

## 4. Diagnosing Chronic Pain

Properly diagnosing chronic pain is crucial to the successful management of the condition. In the field of headache research, strict criteria for headaches such as migraines help dictate diagnosis and treatment and facilitate additional inquiry. As previously discussed, chronic pain is currently defined as pain that persists or recurs for more than 3 months, consistent with temporal cutoffs associated with other chronic diseases [29]. The 3-month criterion is a common temporal cutoff for chronic conditions. This allows chronic pain diagnoses to remain consistent with criteria of many other chronic health conditions, which facilitates a uniform measure across clinical practice, health statistics, and academia.

Reaching a timely, accurate diagnosis for chronic pain is important to avoid progression towards a chronic disease. One study examining 180 patients with complex regional pain syndrome (CRPS) found that a long a time between onset and diagnosis was predictive for late recovery and the progression of symptoms [30]. Similarly, data from a cross-sectional study found a median diagnostic delay of eight years for spondyloarthritis, and delayed diagnosis was also associated with worse outcomes and poor treatment responses [31]. Another complex disorder, fibromyalgia, is a challenge for healthcare providers as one study found a mean time to diagnosis as long as 6.42 years, with older patients diagnosed at a slower rate [32].

There have been recent initiatives to develop a more personalized and precision-based approach to chronic pain diagnosis and treatment. For example, one study examined a diagnostic approach that would address specific mechanisms behind “non-specific” chronic low back pain to personalize treatment [33]. Described as a “pain diagnostic ladder”, this approach encourages a classification of chronic low back pain based on its anatomical, pathological, and mechanistic base [34,35,36].

To localize pain anatomically, a proper musculoskeletal (MSK) physical exam and local anesthetic injections may be utilized as diagnostic tools. However, the MSK physical exam has limited localizing value, and local anesthetic injections are an invasive means of deriving diagnostic information. It is valuable to monitor for changes in the quality of the patient’s pain that may suggest persistent changes in central nervous system (CNS) nociceptors, which may decrease the relevancy of identifying a precipitating peripheral cause of pain.

The authors of this study additionally suggest increasing the characterization for pathological “degenerative low back pain”, advocating for distinctions between pathologies that are nociceptive [37], inflammatory [38], neuropathic, or centralized/dysfunctional [39]. Finally, the mechanistic component of pain refers to its cellular mechanisms, which may be utilized to narrow down pharmacological interventions. Examples include utilization of NMDA-antagonists such as ketamine or dual amine uptake inhibitors for allodynia [40]. Chronic pain diagnoses that follow a “pain diagnostic ladder” or similar schematic may allow greater personalization of treatment plans and targeting of patients’ distinct pain etiologies.

## 5. Pain Subclassifications

The classification of pain is vital for the proper treatment of patients, health care policies, statistics, and reimbursement. Neuropathic pain is one example of a condition that has been brought up numerous times as a major epidemiological problem needing systematic classification [41]. In 2018, the World Health Organization (WHO) released the first ever systematic classification of chronic pain diagnoses as part of the ICD-11. Given the high global prevalence of chronic pain, affecting over 30% of the world population [18], the development of a systematic classification for chronic pain facilitates the collection of thorough epidemiological data. These subclassifications have been used to report health care statistics from January 2022 and onwards. The classification system discussed below is intended to apply to specialized pain management and primary care alike.

When diagnosing chronic pain, it is important to distinguish chronic primary pain from chronic secondary pain syndromes (Table 1). Chronic primary pain is defined by the IASP as pain in one or more anatomical regions that is characterized by significant emotional distress or functional disability, and which is not better explained by an alternative chronic pain subclass [42]. Chronic primary pain syndromes include fibromyalgia, complex regional pain syndrome, chronic primary headache, chronic primary visceral pain such as irritable bowel syndrome, and chronic nonspecific low-back pain. These conditions are precluded by chronic pain that can be characterized as a standalone primary diagnosis.

In contrast, chronic secondary pain syndromes arise from another health condition as the underlying cause. In these conditions, pain may have been a symptom of an underlying illness prior to becoming its own autonomous health condition. The diagnosis of chronic secondary pain syndrome may be prompted when the patient’s pain requires its own care and treatment plan, or when the pain persists despite resolution of the initial underlying illness. It is important to exclude patients who have underlying conditions commonly associated with pain, but do not themselves meet criteria for a co-diagnosis of chronic pain.

Chronic secondary pain is further divided into six subcategories, all of which must still meet the minimum 3-month duration for chronic pain. These subcategories include chronic cancer-related pain, chronic postsurgical or post-traumatic pain, chronic neuropathic pain, chronic headache and orofacial pain, chronic secondary visceral pain, and chronic secondary musculoskeletal pain. Chronic cancer-related pain may be caused by cancerous growth or spread or by chemotherapy or radiation treatment. Meanwhile, pain related to surgical intervention for cancer falls under the chronic postsurgical or post-traumatic pain category. Notably, postsurgical pain often stems from a peripheral neuropathic etiology. Therefore, chronic neuropathic pain is a common co-diagnosis. Chronic daily headache is defined by the International Headache Society (IHS) as “15 or more headache episodes per month for at least three months [43]” Common secondary causes of chronic headache include central nervous system infections, tumors, hematomas, aneurysms, or vasculitis [44,45,46,47,48].

Medication overuse is also an important secondary cause of chronic headaches [49]. This subclass often overlaps with primary headache disorders such as migraines, tension headaches, and cluster headaches as precipitants to medication overuse. This is just one example of how important it is to screen patients for underlying primary disorders before initiating treatment based on the secondary cause. A similar pattern may be seen in chronic secondary visceral and secondary musculoskeletal pain. Patients whose chronic visceral or musculoskeletal pain is idiopathic or has an established functional mechanism should be treated for a primary chronic pain disorder. If it is determined that the patient’s pain is truly secondary in nature, then chronic visceral and musculoskeletal pains may be further characterized by their etiologies, including, but not limited to, mechanical, vascular, or inflammatory mechanisms.

## 6. Somatosensory Assessments

Common chronic pain management seeks to initially rule out any treatable causes of the pain, and then to provide the patient with as high a quality of life as possible [50]. Somatosensory assessments of pain in a clinical setting often use cutaneous stimuli, such as touch and light pressure, or deep pressure stimuli, such as manually inflated cuffs or instruments for pressure. Most commonly, pain thresholds are evaluated by applying cutaneous and deep pressure stimuli to control sites and the sites of reported pain. Research has suggested the use of mechanical stimuli, such as touch or punctuate pressure, are predictive of pain intensity [51].

The current gold standard of chronic pain assessment is based on self-reports of sensory intensity. This can be used via categorical scales, numerical rating scales, visual analog scales, and descriptor scales, though numerical scales are the most used. This method relies on patient recall to define temporal features of pain, i.e., the variability of the pain: whether it is intermittent, constant, or changing in intensity. The recommended numerical rating scale asks patients to rank their pain on a 0–10 scale, with 0 as an indication of no pain and 10 equaling the worst possible pain [52].

Current guidelines for assessing somatosensory function in chronically ill patients have been outlined by the German Neuropathic Pain Network (DFNS) [53]. These include measures to evaluate for temporal and conditioned pain and are used to explore facilitators and inhibitors of pain. For young children or patients with limited verbal ability, it has been suggested that the use of a Faces Pain Scale may be a more accurate predictor, in which patients are shown pictures of facial expressions depicting pain and asked for the image they identify their pain with [54]. Quantitative sensory testing (QST) is another developing assessment that can be used to examine thermal (cold, warmth, etc.) and mechanical thresholds (touch and vibration) to characterize peripheral and central mechanisms of pain. This can used to predict propensity to develop chronic pain and sensitivity to treatment effects [55].

Pain biomarkers are a frequently considered method of evaluating chronic pain, as researchers seek specific neuronal activity that defines pain [56]. This accounts for two forms of pain biomarkers: pain selective and pain specific. Pain selective markers are graded neurons, visualized with higher activation during pain but also present in the absence of pain. Pain specific markers are based on an all-or-nothing response, firing only when the pain is present but never in the absence of pain. The challenge in this approach is the difficulty in defining pain specific markers to an exclusive mechanism of pain, as pain often shows activity within many neurons, and the pain biomarkers of a healthy brain may differ from those in a chronically ill patient [57]. Further evaluation of these biomarkers can help facilitate more personalized treatments for chronic pain.

## 7. Imaging and Clinical Prediction Models

Neuroimaging, such as MRIs, may also be used to assess chronic pain. The functional MRI (fMRI) measures changes in blood oxygenation levels and is an indirect indicator of brain activity. Resting state fMRIs are conducted to view the brain activity of a patient in chronic pain without additional external stimuli, to provide a baseline of the brain’s functional connectivity [58]. This may be compared to the brain’s activity when the chronic pain is exacerbated, specifically seeking alterations in brain networking of resting low oscillatory activity. Other methods of assessing cerebral brain flow for chronic pain include PET and arterial spin labelling^53^. Further methods to explore changes in brain networking include diffusion tensor imaging, which evaluates for variations in the structural connectivity of brain regions. Changes in brain structures are another commonly used means to assess chronic pain. Some investigators suggest reductions in gray matter volume or other changes in brain structure may be associated with pain [52].

Multivariate pattern analysis (MVPA) may additionally be used to compare healthy controls to individuals in pain. This requires patterns of brain activity in control versus chronic pain patients to specify brain structure or activity that may be contributing to pain. Over time, MVPA is anticipated to become a more widely acknowledged diagnostic tool to aid in defining prognoses and tailoring treatment to an individual’s brain activity and structure [59]. Neuroimaging may be utilized in evaluating chronic pain as quantitative measurements which don’t require external stimuli, as compared to measurements via stimuli-based abnormalities, such as the EEG. However, these methods are currently not as widely available for use by clinicians [60].

As the MRI assesses spatial resolution, it may be more accurate to combine neuroimaging with measurements of temporal resolutions, such as the EEG or MEG [59]. However, the expense and lack of specificity associated with neuroimaging limits the clinical use of this method, especially when compared to the practicality of self-reports. Further methods of quantifying chronic pain may include genotyping (identifying genetic markers of mechanisms that contribute to pain), pharmacological studies (clinical responses to drugs), and chemical neuroimaging (ligand-based imaging/magnetic resonance spectroscopy). Nonetheless, these methods remain of low clinical utility, as they are both nonspecific and expensive [52].

Several risk factors have been attributed to chronic pain, including socio-economic, psychological, clinical, and biological factors. Prior literature concluded that to prevent and reduce the impact of chronic pain, modifiable risk factors (e.g., nutrition, physical activity, and acute pain) need to be addressed [61]. Clinical prediction models for the future development of chronic pain have thus far been an ongoing challenge to the pain research community, especially due to the wide ranges of pain perception. One study examined chronic pain associated with nerve injuries after surgical and medical procedures; it found that the severity of chronic postoperative pain can be predicted by experimental pain assessment prior to surgery, specifically having the endogenous analgesia system tested with diffuse noxious inhibitory control (DNIC) [62]. There are more studies exploring the predictive value of presurgical psychosocial pain assessment for acute postoperative pain, yet fewer that study biomarkers predicting chronic pain.

## 8. Management of Chronic Pain

Conservative management of pain is generally the first intervention tried by patients when symptoms of pain first arise, and treatment strategies often include avoidance of triggers for pain, physical therapy, and often non-narcotic analgesics. Due to the multifactorial disease process and various parts of pathophysiology yet still unknown with chronic pain, multiple treatment modalities are needed to produce significant pain relief for patients [63,64]. A biopsychosocial approach to multidisciplinary pain management can coexist with use of analgesics, interventions, etc. [65]. Generally, opioids are less effective for chronic non-cancer pain compared to use on a short-term basis and at low doses used for acute postsurgical pain [66]. Besides pharmacological treatment for chronic pain, there have also been studies concluding there is an inverse dose–response association between physical activity and chronic pain [67].

Recent randomized controlled trials have also been more frequent in examining treatments tailored towards the biopsychosocial approach to managing chronic pain. For example [68], one clinical trial studying patients with chronic lower back pain found that sensorimotor retraining interventions resulted in improved pain intensity. Retraining focused on altering how patients thought about their pain, processed sensory information, and moved their back during activities. However, one weakness of this trial was that there was no double-blindingor the clinicians in either of the treatment arms, which would have reduced bias. Another meta-analysis [69] of individual cognitive functional therapy, compared to group-based exercise intervention, found no significant difference in pain intensity. Patients who underwent individualized cognitive functional therapy, however, reported greater long term improvements in disability associated with chronic pain.

Pharmacological treatment of chronic pain conditions primarily associated with nociception include acetaminophen, non-steroidal anti-inflammatory drugs (NSAIDs) and other neuroactive drugs for patients with neuropathy or central sensitization. Education, physical exercise, and cognitive behavioral therapy have been shown to be effective for almost any type of pain [70]. Neuromodulation techniques for pain management have also been on the rise [71,72,73,74]. Local anesthetics have also been used beyond intraoperative anesthesia and analgesia for treatment of both acute and chronic pain conditions [75,76]. There needs to be a strong therapeutic alliance between clinicians and patients, from diagnosing and classifying chronic pain to correctly imaging, assessing, and ultimately treating the disease.

Antidepressants have also been employed as an off-label treatment for chronic pain conditions such as fibromyalgia, neuropathic pain, and musculoskeletal pain. One meta-analysis [77] examining several antidepressants for chronic pain outcomes found duloxetine as the only option with effectiveness as a pain reliever. Medical cannabis is another treatment that has gained more popularity to treat chronic pain more refractory to other pharmacological and interventional methods. Thirty-two trials [78] consisting of over five thousand enrolled patients with chronic and cancer-related pain were reviewed and concluded that medical cannabis has utility for pain relief and improved sleep quality. Currently, there is a lack of assessment on the long term effects of medical cannabis, and no previous trial has followed patients more than five and a half months.

Interventional pain management and sometimes even surgical intervention may be indicated for chronic pain with a lumbar spine etiology. For example, therapeutic epidural injections—caudal, lumbar, interlaminar, and transforaminal—have been utilized to manage chronic lower back pain secondary to disc herniation. A systematic review [79] showed stronger evidence for short-term effects in alleviating pain and disability compared to long-term effects. There is a lack of trials with one year follow-up in patients who underwent epidural injection under fluoroscopy.

Radiofrequency ablation (RFA) is another interventional procedure using heat to treat chronic lower back pain associated with lumbar facet and sacroiliac joints. One review of eleven RCTs showed evidence for RFA as an effective short-term treatment but less so for the treatment of intervertebral (discogenic) pain [80]. There has been a recent interest and growing research into discogenic chronic lower back pain being treated with intraosseous basivertebral nerve (BVN) ablation. BVNs are thought to be responsible for transmitting pain signals from vertebral end plates often associated with discogenic disease [81]. One study [82] observing patients for two years following BVN ablation showed significant improvement of pain and function compared to the standard care arm of the trial. While this relatively new procedure is considered minimally invasive and safe, there should be a wave of upcoming research into any long term adverse effects such as potential vertebral compression fractures, especially in an elderly population who may already be predisposed to osteoporosis.

## 9. Conclusions

Pain medicine is a multidisciplinary and multimodal approach to help patients manage chronic pain. Over the recent years, the definition of chronic pain has evolved to become less “non-specific” and to help aid in the proper classification of the disease. While the term can often be too generalized when describing a wide array of conditions, the future of chronic pain diagnosis and treatment is becoming more personalized and precision based. More optimized and specific chronic pain diagnoses can help avoid pooling together heterogenous conditions. A further revision of ICD diagnoses for chronic pain is needed to better accomplish this goal.

Millions are affected by chronic pain physically, emotionally, and socially, with a tremendous impact on economic costs. A biopsychosocial assessment is most helpful in understanding all facets of chronic pain. In addition to recognizing the cognitive elements of pain, treatment for chronic pain will comprise pharmacological therapy, physical rehabilitation, and potentially interventional procedures for refractory pain.

Chronic pain also has a significant burden on older populations and is associated with lower socioeconomic status, lost productivity, and disability, along with its functional, emotional, and social impacts. In this review, a range of definitions of acute to chronic pain were outlined with the goal a more mechanistic approach to address the growing understanding of the anatomical and physiological processes involved. Pain subclassifications can assist in distinguishing primary and secondary pain syndromes, which is important in the screening phase prior to initiation of treatment. Proper classification of a patient’s chronic pain can help treat the underlying cause of illness.

There is ongoing research in the field of somatosensory assessments for chronic pain ranging from self-reported measures of sensory intensity to the use of mechanical stimuli, even quantitative sensory testing to assess thermal and mechanical thresholds for pain. Clinical studies for chronic pain need to be more inclusive of feasible solutions. Policy makers and regulators need to be alerted to the economic and personal burden of this widely common disease to better diagnose and classify chronic pain conditions [83]. Additionally, there has been progress in learning more about pain selective and pain specific biomarkers, but there needs to be more research done to correlate them with exclusive mechanisms of chronic pain.

Delayed diagnosis can often lead to worse outcomes for patients with chronic pain, as is often seen with older adults. This increases the importance for correct diagnosis and classification of the disease process. Other imaging techniques like neuroimaging (fMRI) and multivariate pattern analysis (MVPA) help investigate the connections between chronic pain and structural and physiological changes in the brain. There is a need for more interest in developing more reliable clinical prediction models for chronic pain. More studies are also needed to examine biomarkers, which can predispose patients to chronic pain, and better understand related comorbid conditions.

The biopsychosocial model also helps facilitate current treatment modalities for chronic pain, which includes pharmacological treatment, physical therapy, neuromodulation, and neuraxial techniques. With the recent advent of opportunities for artificial intelligence in medicine [84,85,86], computer-aided diagnosis of extremely common chronic pain conditions like lower back pain may help improve diagnosis and treatment rates [87]. Earlier and more accurate diagnosis and classification can pave the road to improved outcomes and treatment for the many suffering from chronic pain. A paradigm shift like this will hopefully help spur efforts for more research into biological and clinical advances for patients suffering from chronic pain.

## Figures and Tables

**Table 1:** Chronic primary pain syndromes vs. chronic secondary pain syndromes.

Chronic primary pain syndromes: *pain in one or more anatomical regions characterized by significant emotional distress or functional disability*	Chronic secondary pain syndromes: *pain arising from another health or underlying medical condition*
Examples: - Fibromyalgia - Complex regional pain syndrome - Chronic primary headache - Chronic primary visceral pain - Nonspecific lower back pain	Subcategories: - Chronic cancer-related pain - Chronic postsurgical/post-traumatic pain - Chronic neuropathic pain - Chronic headache and orofacial pain - Chronic secondary visceral pain - Chronic secondary musculoskeletal pain

## Data Availability

Not applicable.
